# Author Correction: 4D-imaging of drip-line radioactivity by detecting proton emission from ^54m^Ni pictured with ACTAR TPC

**DOI:** 10.1038/s41467-022-33708-9

**Published:** 2022-10-10

**Authors:** J. Giovinazzo, T. Roger, B. Blank, D. Rudolph, B. A. Brown, H. Alvarez-Pol, A. Arokia Raj, P. Ascher, M. Caamaño-Fresco, L. Caceres, D. M. Cox, B. Fernández-Domínguez, J. Lois-Fuentes, M. Gerbaux, S. Grévy, G. F. Grinyer, O. Kamalou, B. Mauss, A. Mentana, J. Pancin, J. Pibernat, J. Piot, O. Sorlin, C. Stodel, J.-C. Thomas, M. Versteegen

**Affiliations:** 1 Centre d’Etudes Nucléaires de Bordeaux Gradignan, UMR 5797 CNRS/IN2P3 - Université de Bordeaux, Gradignan, Cedex France; 2 Grand Accélérateur National d’Ions Lourds, CEA/DRF-CNRS/IN2P3, B.P. 55027, Caen, Cedex France; 3 Department of Physics, Lund University, Lund, Sweden; 4 Department of Physics and Astronomy and National Superconducting Cyclotron Laboratory, Michigan State University, East Lansing, MI USA; 5 IGFAE and Dpt. de Física de Partículas, Univ. of Santiago de Compostela, Santiago de Compostela, Spain; 6 Instituut voor Kern- en Stralingsfysica, KU Leuven, Leuven, Belgium; 7 Department of Physics, University of Regina, Regina, SK Canada; 8 RIKEN Nishina Center, Wako, Saitama Japan

**Keywords:** Experimental nuclear physics, Theoretical nuclear physics, Techniques and instrumentation

**Correction to**: *Nature Communications* 10.1038/s41467-021-24920-0, published online 10 August 2021

The original version of this Article contained an error in Fig. 1, in which the 3386 gamma line de-exciting the 10+ isomer should end at the 6+ state (and not the 4+ state).

The correct version of Fig. 1 is: 
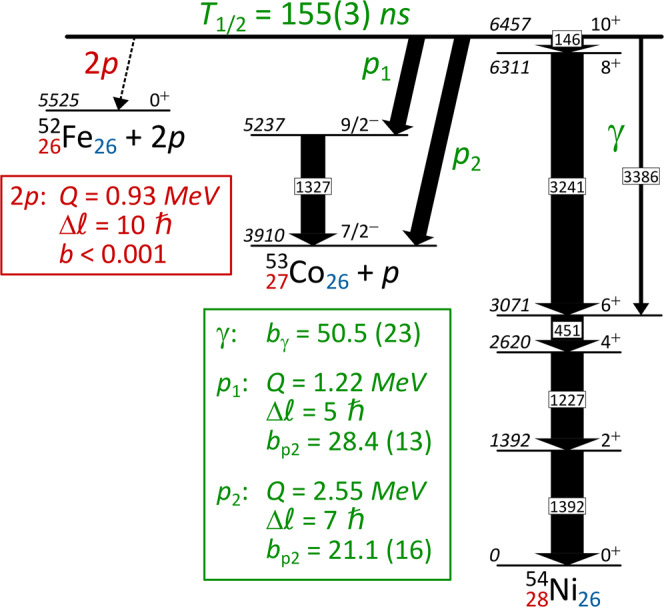


which replaces the previous incorrect version:
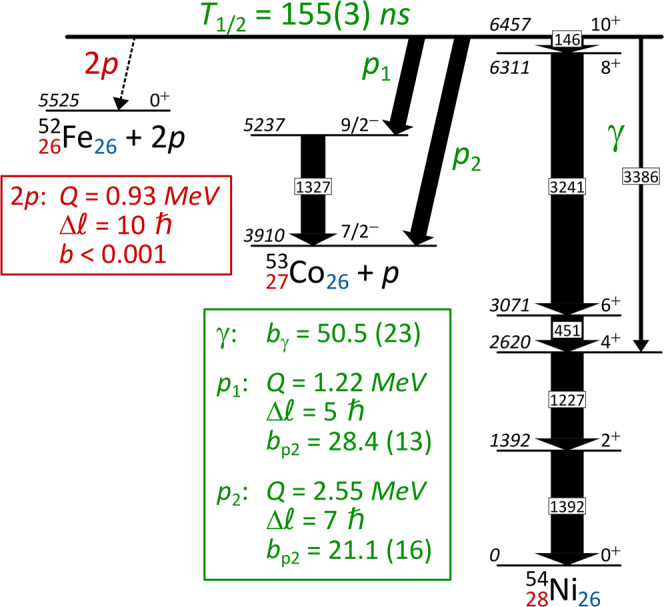


This has been corrected in both the PDF and HTML versions of the Article.

