# Supplementary material for: 4D-imaging of drip-line radioactivity by detecting proton emission from 54mNi pictured with ACTAR TPC
Source: Nat Commun. 2021 Aug 10;12:4805. doi: 10.1038/s41467-021-24920-0 (PMC8355099; doi:10.1038/s41467-021-24920-0)
Supplement: Supplementary file 1 — Supplementary Information [file 41467_2021_24920_MOESM1_ESM.pdf]

Description of Additional Supplementary Figure

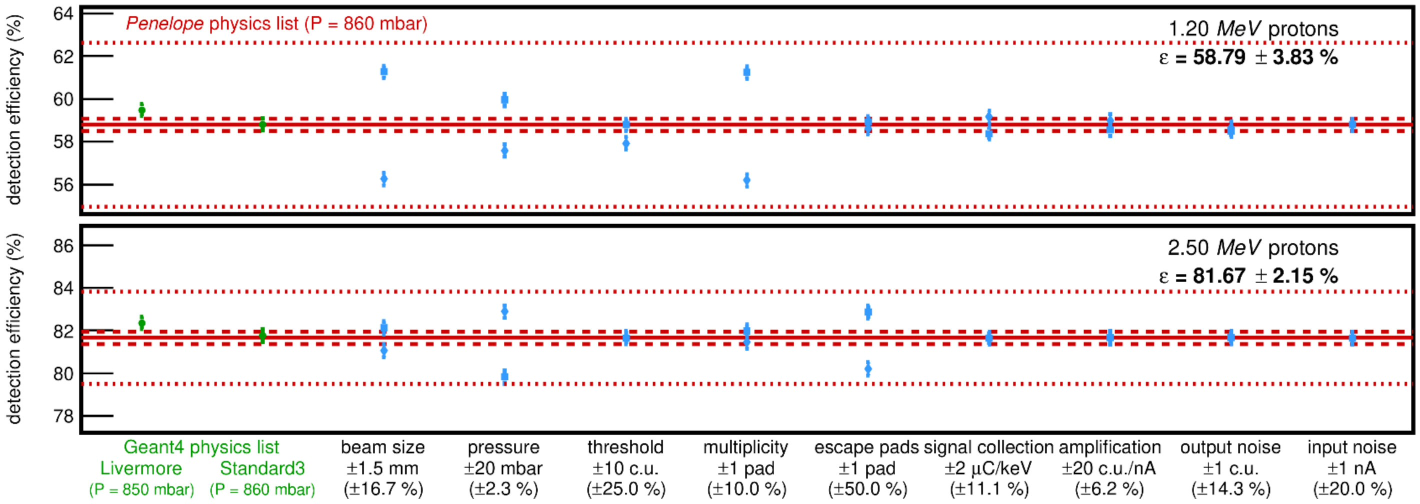

Supplementary Figure 1. Plot showing the contributions of simulation parameters to the uncertainties of the detection efficiencies
