# Supplementary material for: 4D-imaging of drip-line radioactivity by detecting proton emission from 54mNi pictured with ACTAR TPC
Source: Nat Commun. 2021 Aug 10;12:4805. doi: 10.1038/s41467-021-24920-0 (PMC8355099; doi:10.1038/s41467-021-24920-0)
Supplement: Supplementary file 3 — Description of Additional Supplementary Files [file 41467_2021_24920_MOESM3_ESM.pdf]

Description of Additional Supplementary Files

File Name: Supplementary Movie 1
Description: a movie showing the principle of the 3D+decay time imaging with ACTAR TPC.

File Name: Supplementary Movie 2
Description: a movie showing the experimental distributions construction with real events.
